# Linear Six-Carbon Sugar Alcohols Induce Lysis of *Microcystis aeruginosa* NIES-298 Cells

**DOI:** 10.3389/fmicb.2022.834370

**Published:** 2022-04-12

**Authors:** Jaejoon Jung, Ye Lin Seo, Sang Eun Jeong, Ju Hye Baek, Hye Yoon Park, Che Ok Jeon

**Affiliations:** ^1^Department of Life Science, Chung-Ang University, Seoul, South Korea; ^2^Nakdonggang National Institute of Biological Resources, Sangju, South Korea; ^3^National Institute of Biological Resources, Incheon, South Korea

**Keywords:** cyanobacterial bloom, mannitol, six-carbon sugar alcohol, cell lysis, outer membrane vesicle

## Abstract

Cyanobacterial blooms are a global concern due to their adverse effects on water quality and human health. Therefore, we examined the effects of various compounds on *Microcystis aeruginosa* growth. We found that *Microcystis aeruginosa* NIES-298 cells were lysed rapidly by linear six-carbon sugar alcohols including mannitol, galactitol, iditol, fucitol, and sorbitol, but not by other sugar alcohols. Microscopic observations revealed that mannitol treatment induced crumpled inner membrane, an increase in periplasmic space, uneven cell surface with outer membrane vesicles, disruption of membrane structures, release of intracellular matter including chlorophylls, and eventual cell lysis in strain NIES-298, which differed from the previously proposed cell death modes. Mannitol metabolism, antioxidant-mediated protection of mannitol-induced cell lysis by, and caspase-3 induction in strain NIES-298 were not observed, suggesting that mannitol may not cause organic matter accumulation, oxidative stress, and programmed cell death in *M. aeruginosa*. No significant transcriptional expression was induced in strain NIES-298 by mannitol treatment, indicating that cell lysis is not induced through transcriptional responses. Mannitol-induced cell lysis may be specific to strain NIES-298 and target a specific component of strain NIES-298. This study will provide a basis for controlling *M. aeruginosa* growth specifically by non-toxic substances.

## Introduction

Excessive proliferation of harmful cyanobacteria, referred to as cyanobacterial blooms or algal blooms, which mainly occurs with high nutrients (nitrogen and phosphorus), high temperatures, and high residence time, causes serious water quality deterioration such as scum formation, unpleasant odor and taste, hypoxia, and mass fish death in aquatic ecosystems (Codd, 2000; Jankowiak et al., 2019). Further, many harmful cyanobacteria produce toxic compounds such as microcystin and nodularin that can be lethal to animals and humans (Falconer, 1996; van Apeldoorn et al., 2007). Therefore, cyanobacterial blooms are a serious global concern, and their prevention and control are of increasing importance for protecting freshwater sources as well as animal and human health (Qin et al., 2015; Zhao et al., 2019). Various cyanobacteria including *Microcystis*, *Anabaena*, *Aphanizomenon*, *Lyngbya*, *Cylindrospermopsis*, *Nodularia*, *Oscillatoria*, *Trichodesmium*, and *Planktothrix* are known to be associated with cyanobacterial blooms (Kruskopf and Du Plessis, 2006; Westberry and Siegel, 2006; Briand et al., 2008; Ploug, 2008; Sharp et al., 2009; Al-Tebrineh et al., 2012). Among these, *Microcystis aeruginosa* is a predominant and infamous cyanobacterium in most cyanobacterial blooms and produces microcystin, which is known to cause liver toxicity and neurotoxicity in humans (Ozawa et al., 2005; Hu et al., 2016).

One of the ways to prevent cyanobacterial bloom occurrence is to reduce the nutrient contents, the main cause of cyanobacterial blooms, in the water body (Xu et al., 2015). Various physical, chemical, and biological approaches have been used to control or suppress existing cyanobacterial blooms (Lawton et al., 1998; Wu et al., 2012; Fan et al., 2013; Visser et al., 2016; Wu et al., 2016; Yu et al., 2017). Physical methods such as mechanical removal, mixing, and ultrasound are difficult to use for large water bodies and consume large amounts of energy (Lawton et al., 1998; Wu et al., 2012; Visser et al., 2016). Chemical agents such as copper algaecides, ferric salt flocculants, clay flocculants, and glyphosate have been relatively widely used for controlling cyanobacterial blooms, but can be harmful to other living things and result in secondary pollution (Fan et al., 2013; Wu et al., 2016; Yu et al., 2017). As alternatives to physicochemical approaches, biological methods using antagonistic organisms or microorganism- or plant-derived natural compounds specifically targeting cyanobacteria, particularly *M. aeruginosa*, have attracted much attraction (Van Wichelen et al., 2016; Lee et al., 2020). For instance, Lee et al. (2020) reported that amentoflavone derived from the plant, *Selaginella tamariscina*, showed a selective killing effect on *M. aeruginosa*.

During the investigation of interactions between *M. aeruginosa* and symbiotic heterotrophic bacteria, a mutation in a carbon metabolism-related-genes of a symbiotic bacterium showed a significant effect on *M. aeruginosa* growth. We hypothesized that organic carbons might have different effects on *M. aeruginosa* growth and we tested the effects of certain organic compounds on the growth of *M. aeruginosa* NIES-298. Unexpectedly, we found that mannitol, considered a beneficial compound with antioxidant or cell-protective effects in organisms, induced rapid cell lysis in *M. aeruginosa* NIES-298 specifically (Smirnoff and Cumbes, 1989; Dianawati et al., 2012). Therefore, in this study we investigated the mannitol-induced cell lysis in *M. aeruginosa* using various tests and microscopic observations. These results can provide some insights in *M. aeruginosa* control.

## Materials and Methods

### Strains and Culture Conditions

*M. aeruginosa* NIES-298, *M. aeruginosa* PCC 7806, *M. aeruginosa* NIBR 18, *Synechococcus* sp. KCTC AG20470, *Anabaena* sp. FBCC 010003, and *M. aeruginosa* N452 were obtained from the National Institute for Environmental Studies (Japan), Pasteur Culture Collection of Cyanobacteria (France), National Institute of Biological Resources (South Korea), Korean Collection for Type Cultures (South Korea), Freshwater Bioresources Culture Collection (South Korea), and Prof. Woojun Park’s laboratory at Korea University, respectively. Strains NIES-298 and PCC 7806 are axenic strains and others are xenic cultures. All experiments were performed with strain NIES-298, unless specified otherwise. All cultures were routinely grown in 250-ml air-filtered cell culture flasks (SPL Life Science, Korea) containing 100 ml BG-11 medium (Sigma-Aldrich, United States) at 25°C under 40 μmol photons m^–2^⋅s^–1^ with a 12 h/12 h light/dark cycle and were maintained by transferring 2% (v/v) of cultures to fresh BG-11 medium every 3 weeks. All experiments used the exponentially growing cells, approximately 15 days after their transfer according to the growth curve. All treatments were performed at 3 h of the light cycle to ensure high metabolic activity (Straub et al., 2011).

### Effects of Various Organic Compounds on *Microcystis aeruginosa* Growth

To assess the effects of various organic compounds (glucose and fructose as basic carbon sources; pyruvate, gluconate, and mannitol) and yeast extract as a known death-inducer of *Synechococcus* (Christie-Oleza et al., 2017) on *M. aeruginosa* growth, each organic compound was added to 30 ml of strain NIES-298 culture in a 60 ml culture bottle with a filter cap allowing aeration (SPL Lifescience, 70325) to a final concentration of 50 μM [0.1% (w/v) for yeast extract] and the culture bottles were incubated at 25°C under 40 μmol photons m^–2^⋅s^–1^ with a 12 h/12 h light/dark cycle, and the growth of *M. aeruginosa* was monitored by measuring optical density (OD, 680 nm) using a microplate reader (Synergy™ HTX, Biotek, United States). Because mannitol had a clear growth inhibition effect on strain NIES-298, the effects of sugar alcohols (glycerol, erythritol, inositol, iditol, fucitol, galactitol, sorbitol, mannitol, and maltitol) depending on their chemical structures as well as of different mannitol concentrations (0–100 μM) on *M. aeruginosa* growth were also assessed using the same procedure.

Because *M. aeruginosa* dwells with other bacteria in the natural freshwater condition, it was tested if mannitol could induce cell lysis of *M. aeruginosa* in the presence of other bacteria. *Pseudomonas* sp. MAE1-K is a symbiotic bacteria isolated from xenic *M. aeruginosa* KW culture. MAE1-K was inoculated into *M. aeruginosa* NIES-298 culture to be 10^7^ cells/ml to axenic. The co-cultures of *M. aeruginosa* NIES-298 and *Pseudomonas* sp. MAE1-K were treated with 50 μM mannitol and the cell lysis of *M. aeruginosa* NIES-298 was assessed.

The effect of light intensity on the mannitol-induced cell lysis was assessed by culturing *M. aeruginosa* NIES-298 treated with 50 μM mannitol under continuous dark, 40, 80, and 160 μmol photons m^–2^⋅s^–1^ with a 12 h/12 h light/dark cycle at 25°C.

Chlorophyll *a* concentration was also measured using an acetone extraction method, for the growth assessment (Inskeep and Bloom, 1985). For measuring the chlorophyll *a* and organic matter released from cells, 1 ml of *M. aeruginosa* culture was centrifuged for 10 min at 13,300 × *g* and the supernatant was filtered using a 0.45 μm PVDF syringe filter (Millipore, United States). The concentrations of chlorophyll *a* and organic matter in the filtrates were determined *via* the acetone extraction and absorbance at 254 nm (A_254_) (Weishaar et al., 2003), respectively. The effects of mannitol on the growth of other cyanobacterial strains (strains PCC 7806, NIBR 18, N452, KCTC AG20470, and FBCC 010003) were tested with 50 μM mannitol as described above and assessed by measuring OD (680 nm).

#### Measurement of Extracellular Mannitol Concentration

The mannitol concentrations in the supernatants of *M. aeruginosa* cultures at 0, 2, 12, and 24 h after 50 μM mannitol treatment were measured using an Agilent Infinity 1290 UHPLC equipped with an Agilent Infinity Lab Poroshell 120 HILIC-Z column (2.1 mm ×100 m, 2.7 μm) at 35°C coupled with an Agilent 6550 QTOF mass spectrometer (LC-QTOF-MS). Raffinose (2 μM) was used as the internal standard to normalize the peak intensity of each sample. Water containing 0.3% ammonium acetate (A) and acetonitrile (B) were used as mobile phases at a flow rate of 0.4 ml/min with the following gradient: 0–1 min 87% B, 1–5 min 50% B, and 5–7 min 87% B. The mass spectrometry was operated with electrospray ionization under the following operation parameters: polarity, negative; gas temperature, 250°C; nebulizer, 35 psig; and MS range, 20–1,000 m/z.

#### Measurement of Caspase Activity and Oxidative Stress

Because caspase induction has been considered a representative programmed cell death response due to oxidative stresses (Bhattacharjee and Mishra, 2020), the caspase activity of *M. aeruginosa* NIES-298 cells was assessed using a caspase-3 activity kit (Biovision, United States), according to the manufacturer’s instructions. Briefly, *M. aeruginosa* cells were treated with 25 and 50 μM mannitol and 1 mM H_2_O_2_ (as a positive control) and were incubated under the same culture conditions described above. Cells were lysed using a lysis buffer and incubated with the caspase-3 substrate, DEVD-AFC (7-amino-4-trifluoromethyl coumarin), at 37°C for 1 h. Fluorescence was measured at 404 nm excitation and 505 nm emission and was normalized to the *OD* values at 680 nm. To assess oxidative stress generation by mannitol treatment, 30 ml of NIES-298 cell cultures in 60-ml air-filtered culture bottles were treated with superoxide dismutase (SOD, 50 U⋅ml^–1^), catalase (50 U⋅ml^–1^), and glutathione (1 mM), along with mannitol (50 μM), and their growth was assessed by measuring OD (680 nm).

### Observation of Morphological Changes Using Microscopy

Morphological changes in *M. aeruginosa* cells by mannitol treatment were observed using differential interference contrast microscopy (DICM; Carl Zeiss Axio Scope.A1, Germany), scanning electron microscopy (SEM; Zeiss Supra 55VP, Germany), and transmission electronic microscopy (TEM; Talos L120C, Czech). For electron microscopic analyses, cells were primarily fixed overnight at 4°C using 2.5% (w/v) glutaraldehyde, rinsed three times with 0.05 M sodium cacodylate buffer (pH 7.4), and post-fixed with 1% (w/v) osmium tetroxide. The fixed cells were dehydrated in an increasing ethanol series (30 min each in 30, 50, 70, 80, 90, and 100% ethanol). For TEM analysis, the fixed cells were embedded in Spurr’s resin, sectioned, stained, and examined by TEM (Spurr, 1969). For SEM analysis, the fixed cells were dried with a critical point dryer using liquid carbon dioxide, sputter-coated with gold, and then examined with SEM (Jeon et al., 2003).

### Transcriptomic Analysis

To investigate mannitol-induced lysis of *M. aeruginosa* NIES-298 cells at the transcriptional level, total RNA was extracted from *M. aeruginosa* NIES-298 cells treated with 25 and 50 μM mannitol for 10 min using a RNeasy Mini kit (Qiagen, Germany). rRNA was removed from the total RNA and the resulting mRNA was sequenced on an Illumina Hiseq 4000 platform at Macrogen (Korea). Low-quality sequencing reads were removed using Sickle (version 1.33)^1^ and high-quality mRNA reads were mapped onto the complete genome of *M. aeruginosa* NIES-298 (CP046058–9; sequenced by our study group) using Burrows-Wheeler Aligner (BWA) software package (version 0.7.17-r1188) (Li and Durbin, 2010). Gene expression levels were calculated as reads per kilobase of gene per million mapped reads (RPKM). Transcriptomic data was deposited to NCBI Bioproject with the accession number PRJNA691993.

### Statistical Analysis

All tests were performed in triplicate. The results were statistically analyzed using unpaired Student’s *t*-test available in R software (version 4.1.0) and were expressed as means ± standard deviations. Statistical significances of the results were calculated compared to the control group at the same incubation time and were indicated with *p*-values < 0.05 (*), < 0.01 (**), or < 0.001 (***).

## Results and Discussion

### Effects of Linear Six-Carbon Sugar Alcohols on the Growth of *Microcystis aeruginosa* NIES-298

**FIGURE 1:**
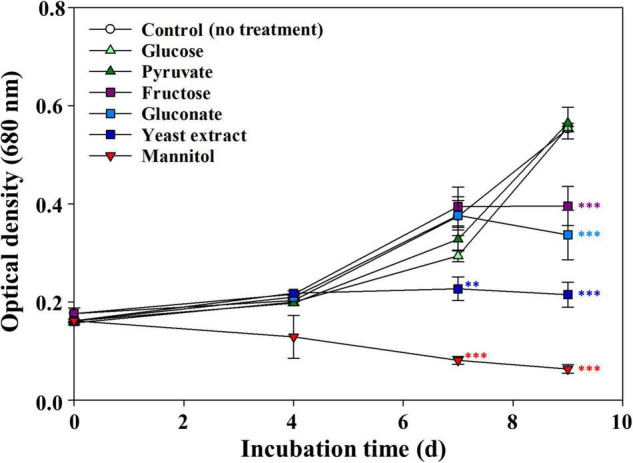
Effects of various organic compounds on the growth of *Microcystis aeruginosa* NIES-298. After adding organic compounds (50 μM), the cultures were incubated at 25°C under 40 μmol photons m^–2^⋅s^–1^ with a 12 h/12 h light/dark cycle. Data are indicated as mean values ± standard deviations. Statistical significances depending on organic compounds were calculated compared to the control group (no treatment) and their significant differences were indicated with different colors. **p < 0.01; ***p < 0.001.

We assessed the effects of various organic compounds on the growth of axenic *M. aeruginosa* NIES-298 (hereinafter *M. aeruginosa*) and found that their effects were quite different depending on the organic compounds used (Figure 1). *M. aeruginosa* cultures supplemented with glucose and pyruvate displayed growth profiles similar to the control cultures (no addition of organic compounds), whereas the growth of cultures supplemented with fructose, gluconate, or yeast extract was shown to be significantly inhibited after 7 days. The growth inhibition of *M. aeruginosa* by yeast extract was partially consistent with a previous report showing that yeast extract accelerated the death of *Synechococcus* sp. WH78909 cells (Christie-Oleza et al., 2017).

**FIGURE 2:**
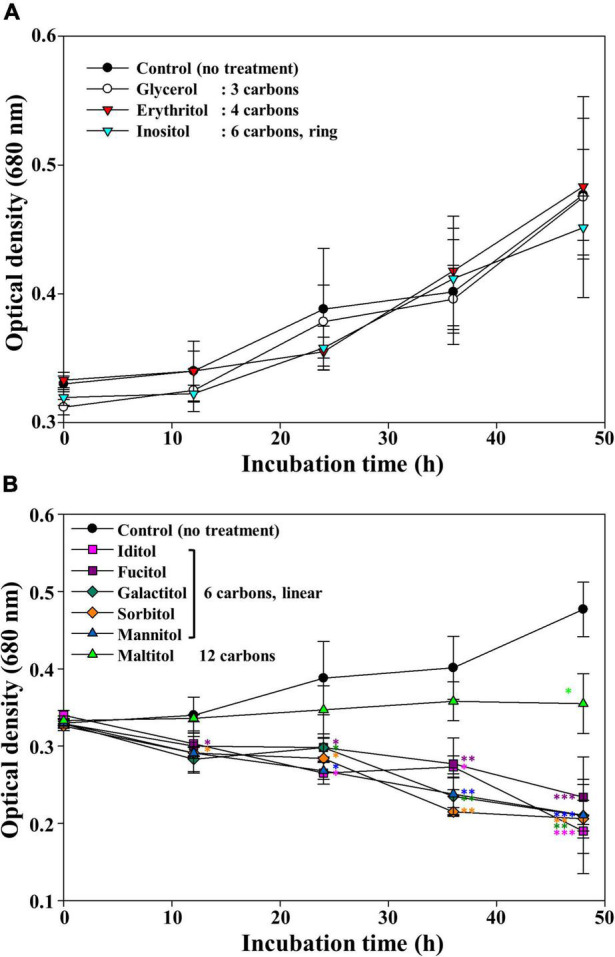
The growth of *Microcystis aeruginosa* NIES-298 treated with sugar alcohols without any effect **(A)** and with inhibiting effect on the growth **(B)**. After adding sugar alcohols (50 μM), the cultures were incubated at 25°C under 40 μmol photons m^–2^⋅s^–1^ with a 12 h/12 h light/dark cycle. Numbers in the parenthesis indicate the carbon numbers of sugar alcohols. Data are indicated as mean values ± standard deviations. Statistical significances depending on sugar alcohols were calculated compared to the control group (no treatment) and their significant differences were indicated with different colors. *p < 0.05; **p < 0.01; ***p < 0.001.

It has been reported that mannitol is not toxic to many other cyanobacteria, marine and freshwater microalgae (e.g., *Synechococcus*, *Emiliania huxleyi*, and *Monoraphidium griffithii*, respectively); in contrast, some eukaryotic microalgae can produce mannitol and use it as a storage compound (Obata et al., 2013; Jacobsen and Frigaard, 2014; Yee, 2015). However, in this study, mannitol induced a remarkable decrease in cell density soon after its addition to the cultures. As mannitol is a sugar alcohol with six carbons, we further investigated whether other sugar alcohols could induce *M. aeruginosa* cell death (Figure 2). Glycerol (3 carbons), erythritol (4 carbons), and inositol (6 carbons, but with a ring structure) showed no growth inhibitory effect on *M. aeruginosa*. On the contrary, all linear six-carbon sugar alcohols including galactitol, iditol, fucitol, and sorbitol clearly induced *M. aeruginosa* cell death, similar to mannitol. Maltitol, a 12-carbon sugar alcohol known as 4-*O*-α-glucopyranosyl-D-sorbitol, had a growth inhibitory effect on *M. aeruginosa* though its effect was weaker than those of other linear six-carbon sugar alcohols (maltitol). These results suggest that linear six-carbon sugar alcohol moieties (structures) have a detrimental effect on the growth of *M. aeruginosa* NIES-298. Hereinafter, we performed further studies using mannitol as a representative linear six-carbon sugar alcohol.

**FIGURE 3:**
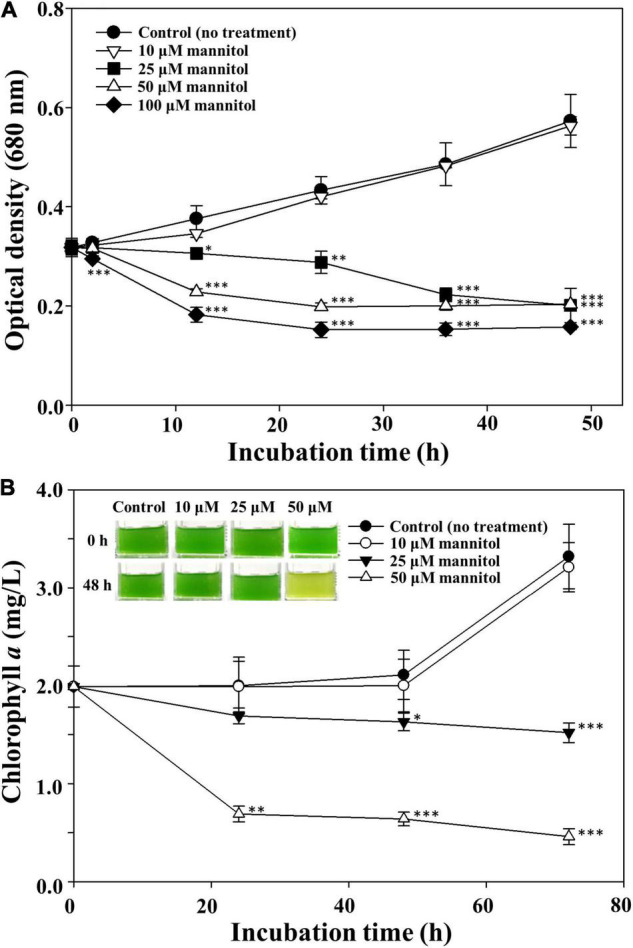
Optical cell density **(A)** and chlorophyll *a* **(B)** profiles of *Microcystis aeruginosa* NIES-298 treated with different concentrations of mannitol. Data are indicated as mean values ± standard deviations. Images showing the color changes of cell cultures after mannitol treatment are also displayed in **(B)**. Statistical significances depending on the mannitol treatments were calculated compared to the control group (no treatment) and their significant differences were indicated with *p*-values < 0.05 (*), < 0.01 (**), or < 0.001 (***).

To assess the threshold mannitol concentration inhibiting the growth of *M. aeruginosa*, different concentrations of mannitol were used to treat *M. aeruginosa* cultures (Figure 3). *M. aeruginosa* growth was very weakly affected by 10 μM mannitol, whereas concentration-dependent growth inhibition of *M. aeruginosa* was observed with more than 25 μM mannitol (Figure 3A), however, the green color of the cultures was maintained for a relatively long time after mannitol treatment (inset of Figure 3B), suggesting that unlike stress-induced death of *M. aeruginosa*, mannitol does not induce chlorophyll bleaching (Park et al., 2010; Alexova et al., 2011; Yang et al., 2017). The effect of light intensity on the cell lysis of *M. aeruginosa* NIES-298 by mannitol was also evaluated and the mannitol-induced cell lysis of *M. aeruginosa* NIES-298 cells occurred similarly even under dark condition regardless of light intensity (Supplementary Figure 1), suggesting that the mannitol-induced cell lysis may proceed through light-independent reactions.

### Possible Mechanisms Involved in Mannitol-Induced *Microcystis aeruginosa* Cell Death

The possible mechanisms by which linear six-carbon sugar alcohols can induce cell death of *M. aeruginosa* were examined. The first possibility is osmotic stress because cyanobacterial cells may be susceptible to osmotic stress (Shapiguzov et al., 2005). However, because *M. aeruginosa* was reported to be tolerant even to 7 g/L NaCl (approximately 120 mM), 25 μM mannitol is unlikely to cause osmotic stress enough to induce cell death (Tonk et al., 2007). The second possibility for cell death induction is the accumulation of reactive oxygen species (Zhou et al., 2018). However, treatments of SOD, catalase, and glutathione failed to rescue the mannitol-induced cell death in *M. aeruginosa* (Supplementary Figure 2), suggesting that oxidative stress may not be the relevant underlying cause. Further, mannitol is known as an antioxidant or compatible solute that protect cells from oxidative and osmotic stresses rather than as an agent inducing oxidative or osmotic stress (Smirnoff and Cumbes, 1989; Sand et al., 2013). The third possibility is the accumulation of organic matter through mannitol metabolism because build-up of organic matter in cultures was reported to be potentially toxic to axenic *Synechococcus* sp. WH7803 (Christie-Oleza et al., 2017). However, mannitol analysis showed that the mannitol concentration in *M. aeruginosa* cultures remained unchanged (Supplementary Figure 3). Genome analysis of *M. aeruginosa* NIES-298 (NZ_CP046058–9) also further indicated that no membrane transport systems and metabolic genes (e.g., mannitol dehydrogenase) were identified for linear six-carbon sugar alcohols. These results suggest that linear six-carbon sugar alcohols may not cause a build-up of organic matter *via* their metabolism, because *M. aeruginosa* cannot metabolize and uptake linear six-carbon sugar alcohols.

**FIGURE 4:**
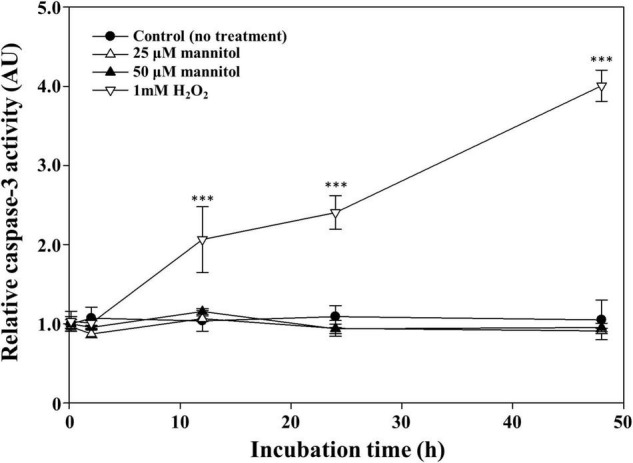
Time course caspase-3 (-like) activity of *Microcystis aeruginosa* NIES-298 cells treated with mannitol and H_2_O_2_ (as a positive control agent). The caspase activities were expressed as arbitrary unit (AU). Data are indicated as mean values ± standard deviations. Statistical significances depending on the treatments were calculated compared to the control group (no treatment) and their significant differences were indicated with *p*-values < 0.001 (***).

Recently, programmed cell death has been proposed as an important cell death mechanism in cyanobacteria including *Anabaena*, *Trichodesmium*, and *Microcystis* (Ning et al., 2002; Berman-Frank et al., 2004; Ding et al., 2012). Programmed cell death is initiated by the induction of caspase, which is resulted in various morphological and biochemical responses (Saraste and Pulkki, 2000; Bhattacharjee and Mishra, 2020). Therefore, the caspase activity of mannitol-treated cells was measured; H_2_O_2_, which is known to induce caspase activity by programmed cell death (Zhou et al., 2018), was used as the positive control. Treatment of *M. aeruginosa* cultures with 1 mM H_2_O_2_ significantly induced caspase-3 (-like) activity at approximately fourfold compared to the untreated cells after 48 h (Figure 4). However, no caspase-3 activity was induced by the treatments with 25 and 50 μM mannitol, suggesting that programmed cell death is not the cause of mannitol-induced cell death in *M. aeruginosa*. Taken together, these results indicate that the cell death mechanism underlying death induction in *M. aeruginosa* by mannitol may not be explained using the existing cell death mechanisms. Therefore, we investigated the morphological changes induced by mannitol treatment in *M. aeruginosa* cells using various microscopic observations to further scrutinize the mannitol-induced cell death process.

### Morphological Changes Induced by Mannitol *Treatment* in *Microcystis aeruginosa* Cells

**FIGURE 5:**
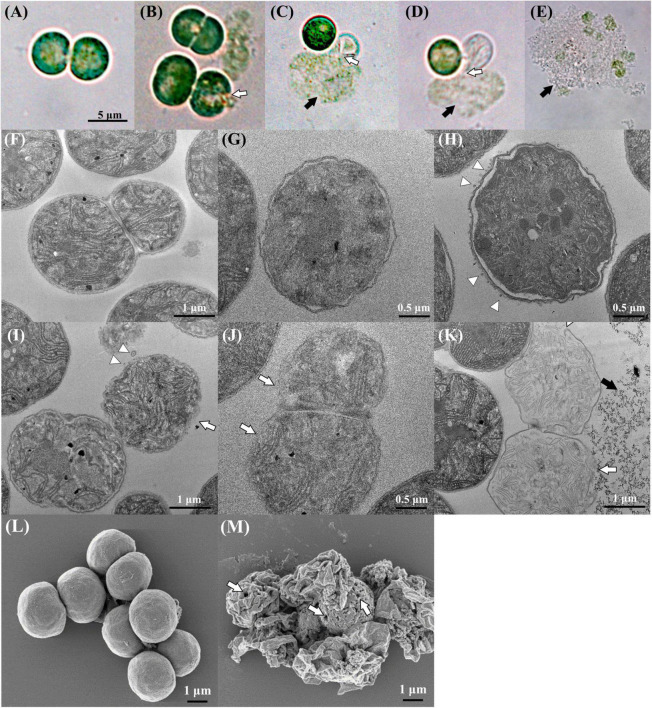
DICM **(A–E)**, TEM **(F–K)**, and SEM **(L,M)** images showing the morphological changes in Microcystis aeruginosa NIES-298 cells upon mannitol treatment (50 μM). Photographs **(A,F,L)** indicate intact cells without mannitol treatment and all mannitol-treated cells **(B–E,G–K,M)** were observed within 2 h (the SEM image in **(M)** was obtained at 2 h after mannitol treatment). After mannitol treatment, the proportion of damaged cells **(F,K)** increased over time. Open arrows, triangles, and closed arrows indicate the disruption of cell walls, outer membrane vesicles, and intracellular matter released from cells, respectively. Open arrows in **(M)** indicate pores formed on cell walls after mannitol treatment.

To investigate the mannitol-induced cell death process, the morphological changes induced by mannitol treatment in *M. aeruginosa* cells were observed by DICM, SEM, and TEM (Figure 5). The DICM images indicated that *M. aeruginosa* cells without mannitol treatment were in a healthy state showing green color as well as round and regular shapes (Figure 5A). TEM and SEM images also showed that mannitol-untreated cells had round and regular shapes without any cell wall damages (Figures 5F,L). In particular, mannitol-untreated cells were shown to have intact and normal cell walls, cell membranes, thylakoid membranes, and storage granules in a constant state in the TEM images (Figure 5F). However, upon mannitol treatment, the cell walls burst rapidly in 10 min (Figure 5B) and intracellular matter including chlorophyll was released out of cells, while maintaining green color (Figures 5C,D); eventually, the green color was bleached (Figure 5E), indicating the death of *M. aeruginosa* cells.

The TEM images displayed more marked morphological changes in *M. aeruginosa* cells upon mannitol treatment. After mannitol treatment, the inner and outer membranes of cell walls were shown to be crumpled with uneven cell shapes, and the periplasmic spaces between inner and outer membranes were increased upon the separation of inner and outer membranes (Figures 5G,H). The thylakoid membranes and granules inside cells became unclear, suggesting the disruption of intracellular structures. Interestingly, many outer membrane vesicles (OMVs) were identified from the outer membranes of cells treated with mannitol (no OMV was detected in mannitol-untreated cells), and eventually, the outer membranes of the cell walls disappeared (Figures 5H–J), probably leading to cell wall disruption and intracellular matter release (Figure 5K). SEM images taken after 2 h of mannitol treatment showed that the mannitol-treated cells were shrunken, probably due to cell wall disruption and intracellular matter release, and that there were some pores on the cell walls probably leading to the intracellular matter release (Figure 5M). These results suggest that mannitol may induce the increase in periplasmic space and formation of OMV, leading to cell wall disruption and intracellular matter release. H_2_O_2_ treatment is also reported to cause *M. aeruginosa* cell shrinkage, but not to cause the perforation of cell walls (Zhou et al., 2020), suggesting that mannitol and H_2_O_2_ induce *M. aeruginosa* cell death *via* different processes.

**FIGURE 6:**
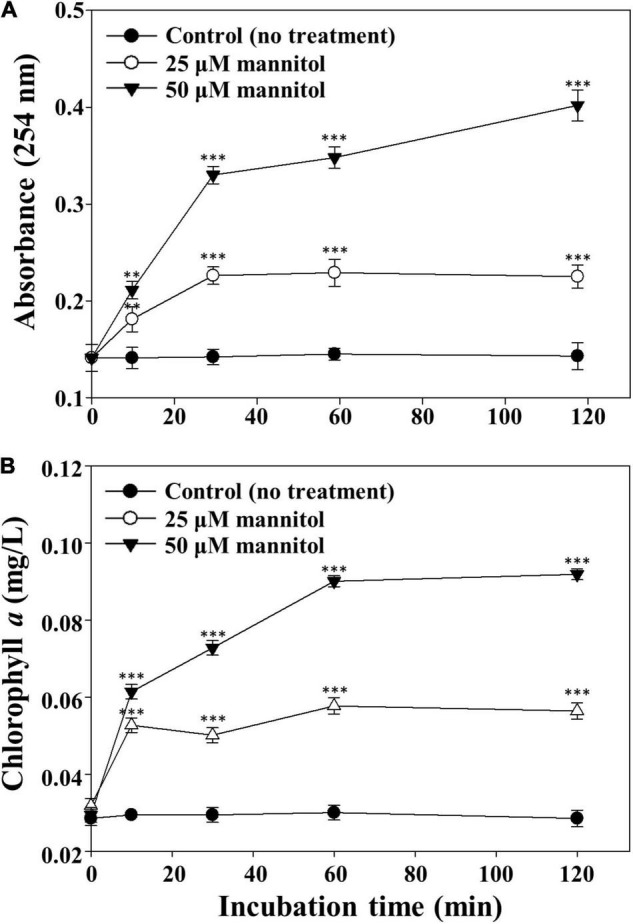
Concentration profiles of intracellular organic matter **(A)** and chlorophyll *a* **(B)** released from *Microcystis aeruginosa* NIES-298 cells treated with mannitol. After mannitol treatment, cell cultures were filtered using a 0.45-μm syringe filter and the concentrations of organic matter (254 nm) and chlorophyll *a* in the filtrates were measured. Data are indicated as mean values ± standard deviations. Statistical significances depending on the mannitol treatments were calculated compared to the control group (no treatment) and their significant differences were indicated with *p*-values < 0.01 (**) or < 0.001 (***).

Microscopic observations of *M. aeruginosa* cells suggest that the disruption of cell walls leading to the release of intracellular matter may occur very rapidly by mannitol treatment. Therefore, the contents of organic matter and chlorophyll *a* released from *M. aeruginosa* cells were measured after mannitol treatment (Figure 6). The concentrations of organic matter and chlorophyll *a* increased quickly and in a concentration-dependent manner by mannitol treatment and these increases were completed in 60 min, suggesting that mannitol-induced cell lysis progresses rapidly to cell wall disruption in *M. aeruginosa*. Morphological changes and cell membrane disruption in *M. aeruginosa* have also been reported to be induced by allelopathic biochemicals such as ferulic acid, prodigiosin, and amentoflavone (Matthijs et al., 2016; Wang et al., 2016; Yang et al., 2017; Lee et al., 2020). However, mannitol-induced cell lysis occurred within a short time (minutes to hours), whereas cell lysis by many other allelopathic biochemicals generally occurs over several days.

Based on eukaryotic cell death mechanisms, Zheng et al. (2013) proposed four death modes for cyanobacterial cells, apoptotic-, autophagic-, autolytic, and necrosis-like deaths, to explain the cell death of a cyanobacterial symbiont of *Azolla microphylla* (Zheng et al., 2013). The cyanobacterial cell death modes were mainly classified based on the structural changes in cell walls, cell membranes, peptidoglycan layers, and intracellular contents, as well as the order of cell death progress (Bhattacharjee and Mishra, 2020). Yang et al. (2017) reported that the death of *M. aeruginosa* FACHB-1752 cell by prodigiosin proceeded by a combination of necrotic- and apoptotic-like process (Yang et al., 2017). However, our microscopic observations showed that mannitol-induced lysis of *M. aeruginosa* cells may not be classified into the above-mentioned four cyanobacterial cell death modes and may proceed through a yet unidentified novel cell lysis mode.

Our microscopic observations showed that OMVs that probably led to cell disruption were formed by mannitol treatment. OMV formation, which has been mainly studied in gram-negative bacteria, has been reported from various cyanobacteria, including filamentous freshwater cyanobacteria such as *Anabaena* (Oliveira et al., 2015) and *Cylindrospermopsis* (Zarantonello et al., 2018), and marine cyanobacteria such as *Prochlorococcus*, *Synechococcus* (Biller et al., 2014), and *Synechocystis* (Pardo et al., 2015). Generally, OMV formation is known to increase under stress conditions (Oliveira et al., 2016; Zarantonello et al., 2018). It is also reported that *Prochlorococcus* and *Synechococcus* may use OMVs as a decoy for a protection from phage infection (Biller et al., 2014) and that *E. coli* may use OMV to excrete misfolded proteins (McBroom and Kuehn, 2007). To the best of our knowledge, this is the first report regarding OMV formation in *M. aeruginosa*. It may be difficult to describe the role of OMV in *M. aeruginosa*; however, our microscopic observations indicate that the mannitol-induced lysis of *M. aeruginosa* cells may be associated with OMV formation.

### Transcriptomic Analysis and Strain Specificity of Mannitol-Induced Cell Death

Because mannitol-induced lysis of *M. aeruginosa* cells differs significantly from the previously proposed cell death mechanisms in *M. aeruginosa*, a comparative transcriptomic analysis was performed to determine the mannitol-induced mechanism of *M. aeruginosa* cell lysis. Only eight and ten genes exhibited different expression with more than twofold changes in RPKM values in 25 μM and 50 μM mannitol-treated cells compared to the control (untreated) cells (Supplementary Table 1). In particular, only four and eight genes were upregulated with more than twofold changes in 25 μM and 50 μM mannitol-treated cells, respectively. Proteins such as hypothetical protein, restriction endonuclease, and 3-oxoacyl-ACP synthase that may not be associated with general cell death processes were differentially expressed, whereas SOD genes (D3800_00390 and D3800_14880) were not differentially expressed. These results contrasted markedly with the previous transcriptomic analyses indicating that hundreds of genes related to oxidative stress defense, electron transfer, photosystem, carbon fixation, central metabolic pathways, and microcystin biosynthesis were differentially expressed in *M. aeruginosa* under stress conditions such as oxidative stress and toxic chemicals (Harke et al., 2017; Jiang et al., 2020; Yang et al., 2020; Zhou et al., 2020). Four putative caspase genes (D3800_09480, D3800_10800, D3800_10885, and D3800_18795) were identified from the genome of *M. aeruginosa* NIES-298 (Klemenčič et al., 2015) and these were not differentially expressed under the mannitol-treated conditions (Supplementary Table 2), which was consistent with our result that caspase activity was not induced by mannitol treatment (Figure 4). These transcriptomic results suggest that mannitol-induced lysis of *M. aeruginosa* NIES-298 cells may not proceed through a cell-death cascade associated with the transcriptional responses proposed previously (Zhou et al., 2020).

Because *M. aeruginosa* co-exists with other microorganisms in the natural environment, the effect of mannitol on *M. aeruginosa* was tested in the presence of other species. *Pseudomonas* sp. MAE1-K (CP023641) isolated from a xenic *M. aeruginosa* culture were inoculated into the axenic *M. aeruginosa* NIES-298 culture, followed by mannitol treatment. The results showed that although the lysis effect was slightly weakened, the mannitol-induced cell lysis of *M. aeruginosa* NIES-298 occurred rapidly (Supplementary Figure 4), suggesting that mannitol can induce the cell lysis of *M. aeruginosa* even in the presence of other microbial cells. Mannitol treatment was also applied to various other cyanobacterial strains, including *M. aeruginosa* PCC 7806, *M. aeruginosa* NIBR 18, *M. aeruginosa* N452, *Synechococcus* sp. KCTC AG20470, and *Anabaena* sp. FBCC010003; however, mannitol did not induce cell lysis or death in any of the other tested strains (Supplementary Figure 5), suggesting that mannitol-induced cell lysis is specific to *M. aeruginosa* NIES-298 and that linear six-carbon sugar alcohols may target specific components, encoded by strain NIES-298, probably through a physicochemical interaction.

## Summary

In this study, we found that linear six-carbon sugar alcohols including mannitol, sorbitol, galactitol, iditol, and fucitol induced cell lysis (death) in *M. aeruginosa* NIES-298. The mannitol-induced lysis of *M. aeruginosa* cells occurred rapidly and concentration-dependently and was specific to strain NIES-298. Further, mannitol treatment induced an increase in the periplasmic space of *M. aeruginosa* NIES-298 through separation of its inner and outer membranes and disappearance of the outer membranes probably through OMVs leading to cell wall destruction and release of intracellular matter including chlorophylls. These cell lysis (death) processes were quite different from the previously proposed cell death modes for *M. aeruginosa*. Further, we demonstrated that linear six-carbon sugar alcohols may induce the cell lysis of *M. aeruginosa* NIES-298 through structure-based physicochemical interaction with specific components encoded by strain NIES-298, and not through a cell-death cascade process associated with transcriptional responses.

The high specificity of linear six-carbon sugar alcohols including mannitol to the cell lysis of strain NIES-298 will limit the use as a control agent of algal blooms because algal blooms are not occurred by a single cyanobacterial strain. However, the specificity of linear six-carbon sugar alcohols to strain NIES-298 may be very good merit to develop an environment-friendly agent that controls *M. aeruginosa* specifically without toxicity to other organisms. Therefore, to apply this study to aquatic environments, further research to investigate the relevant cell lysis mechanisms in more detail is necessary. Even though few previous research succeeded mutagenesis of *M. aeruginosa*, genetic manipulation, random mutagenesis, or long-term laboratory evolution under the sub-lethal concentration of mannitol may be able to identify the mechanism of mannitol-induced cell lysis. It will provide a basis for developing novel environment-friendly agents or strategies for the specific control of *M. aeruginosa*, key cyanobacteria that cause algal blooms in freshwater.

## Data Availability Statement

RNA-seq data is publicly available from NCBI BioProject under the accession number of PRJNA691993.

## Author Contributions

JJ, YS, SJ, and CJ designed the research. JJ conducted experiments and performed the main data analysis. YS conducted the main experiments. JB performed LC/MS analysis. SJ and HP conducted data analysis. CJ supervised the study and obtained funding. YS, JJ, and CJ wrote the manuscript. All authors have read and approved the final version of the manuscript.

## Conflict of Interest

The authors declare that the research was conducted in the absence of any commercial or financial relationships that could be construed as a potential conflict of interest.

## Publisher’s Note

All claims expressed in this article are solely those of the authors and do not necessarily represent those of their affiliated organizations, or those of the publisher, the editors and the reviewers. Any product that may be evaluated in this article, or claim that may be made by its manufacturer, is not guaranteed or endorsed by the publisher.

## Footnotes

1
https://github.com/najoshi/sickle
