# Supplementary material for: Linear Six-Carbon Sugar Alcohols Induce Lysis of Microcystis aeruginosa NIES-298 Cells
Source: Front Microbiol. 2022 Apr 12;13:834370. doi: 10.3389/fmicb.2022.834370 (PMC9039742; doi:10.3389/fmicb.2022.834370)
Supplement: Supplementary file 1 [file Data_Sheet_1.DOCX]

**Supplementary** FIGURE **S1.** The effects of light intensity on the cell lysis of *M. aeruginosa* NIES-298 by mannitol (50 μM). Data are indicated as mean values ± standard deviations. Statistical significances depending on the conditions were calculated compared to the control group (40 μmol photons, no mannitol) and showed *p* < 0.001 (***) in all conditions.

**
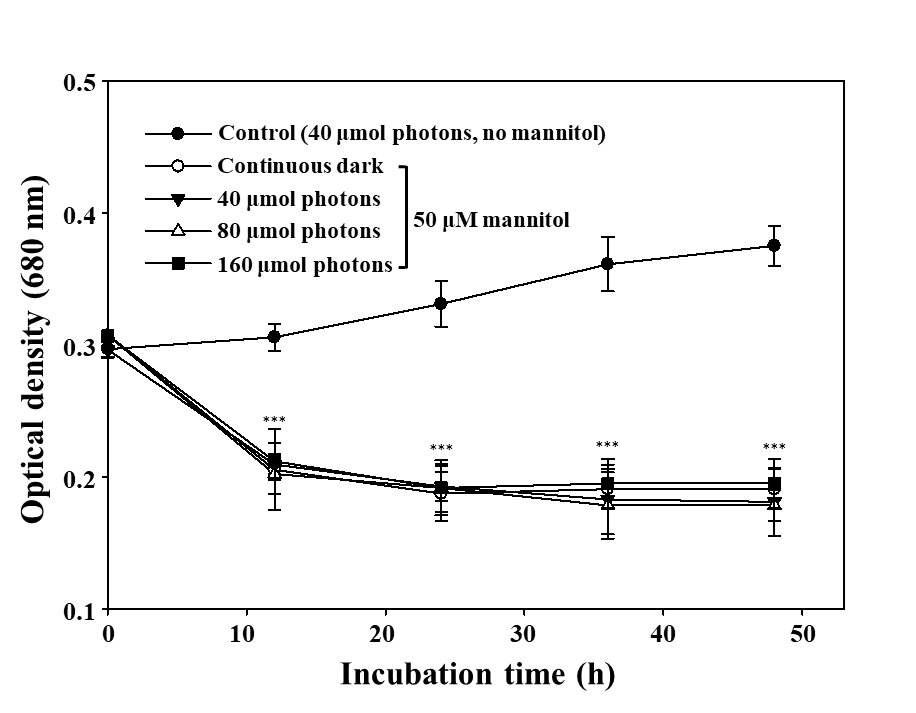
**

**Supplementary** FIGURE **S2.** Effects of superoxide dismutase (SOD, 50 U·ml^-1^), catalase (50 U·ml^-1^), and glutathione (1 mM) on the mannitol (50 μM)-induced cell death of *Microcystis aeruginosa* NIES-298. Statistical significances depending on the treatments were calculated compared to the control group (no mannitol) and showed the same *p* values in all treatments. **, *p* < 0.01; ***, *p* < 0.001.

**
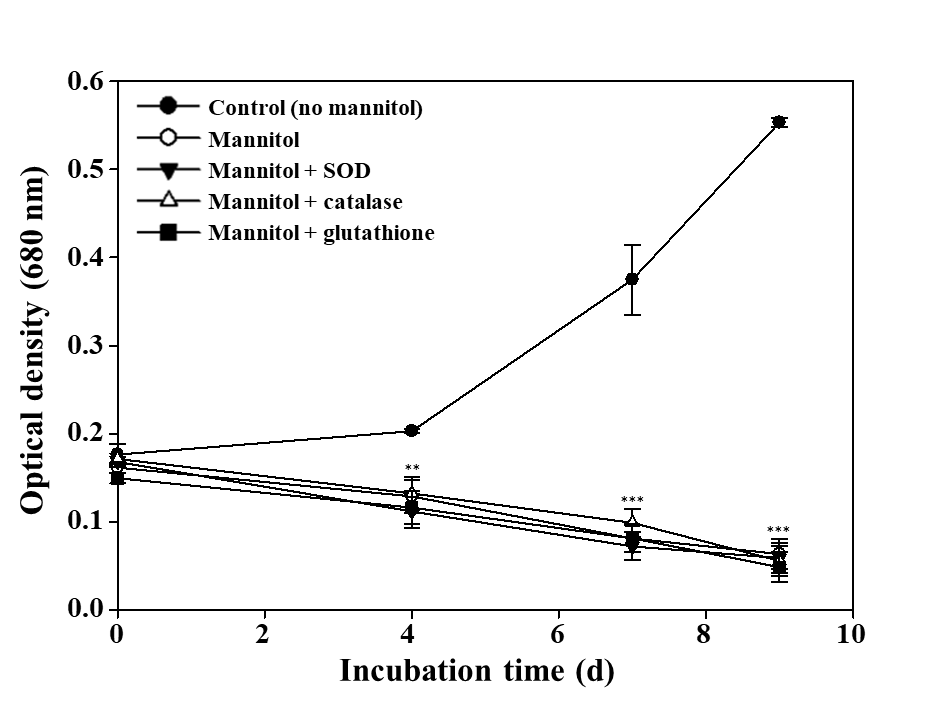
**

**Supplementary** FIGURE **S3.** Mannitol concentrations, measured by LC-QTOF-MS, over incubation time in *M. aeruginosa* cultures supplemented with 50 μM mannitol. Statistical significances over time were calculated compared to the initial time (0 h) and there were no significant differences in all incubation times.


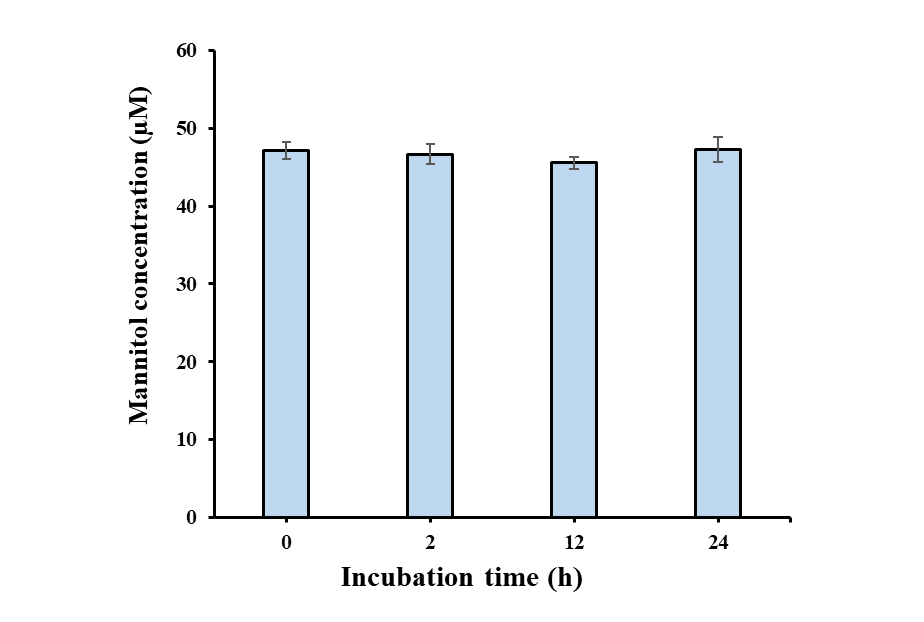


**Supplementary** FIGURE **S4.** Representative pictures showing cell lysis of *M. aeruginosa* NIES-298 cocultured with heterotrophic bacteria isolated from xenic *M. aeruginosa* cultures by mannitol treatment (50 μM).


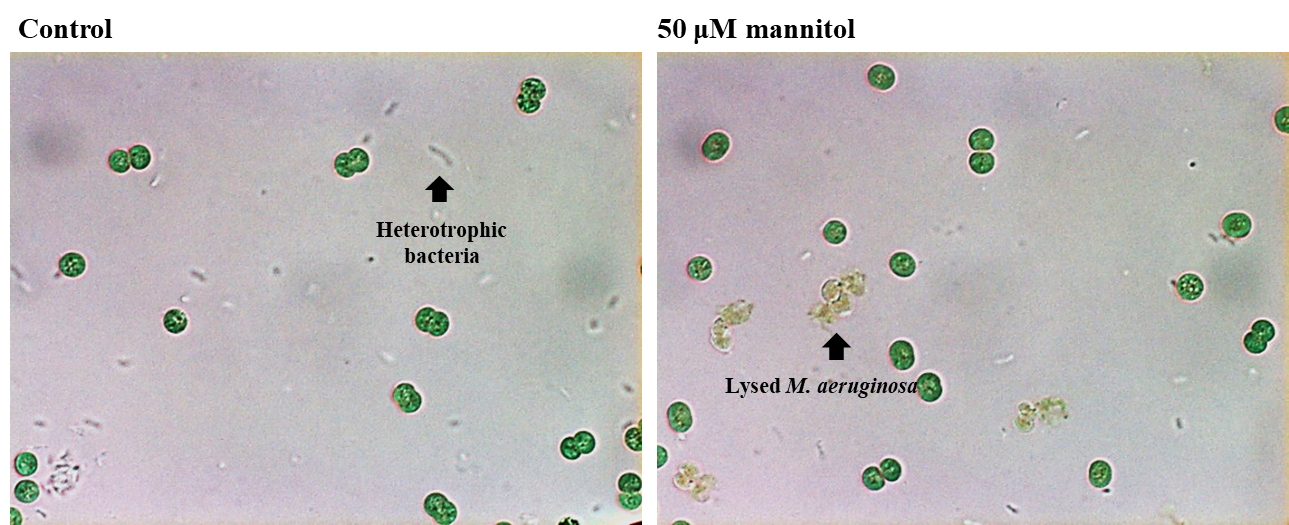


**Supplementary** FIGURE **S5.** Effects of mannitol (50 μM) on the growth of *M. aeruginosa* PCC 7806 (axenic, A), *M. aeruginosa* NIBR 18 (xenic, B), *M. aeruginosa* N452 (xenic, C), and *Synechococcus* sp. KCTC AG20470 (xenic, D). The effect on the growth of *Anabaena* sp. FBCC010003 was not shown because measuring its optical density was impossible due to the aggregation of filamentous cells; however, no cell lysis due to mannitol was not observed. Statistical significances depending on mannitol treatments were calculated compared to the no treatment groups and there were no significant differences in all strains.


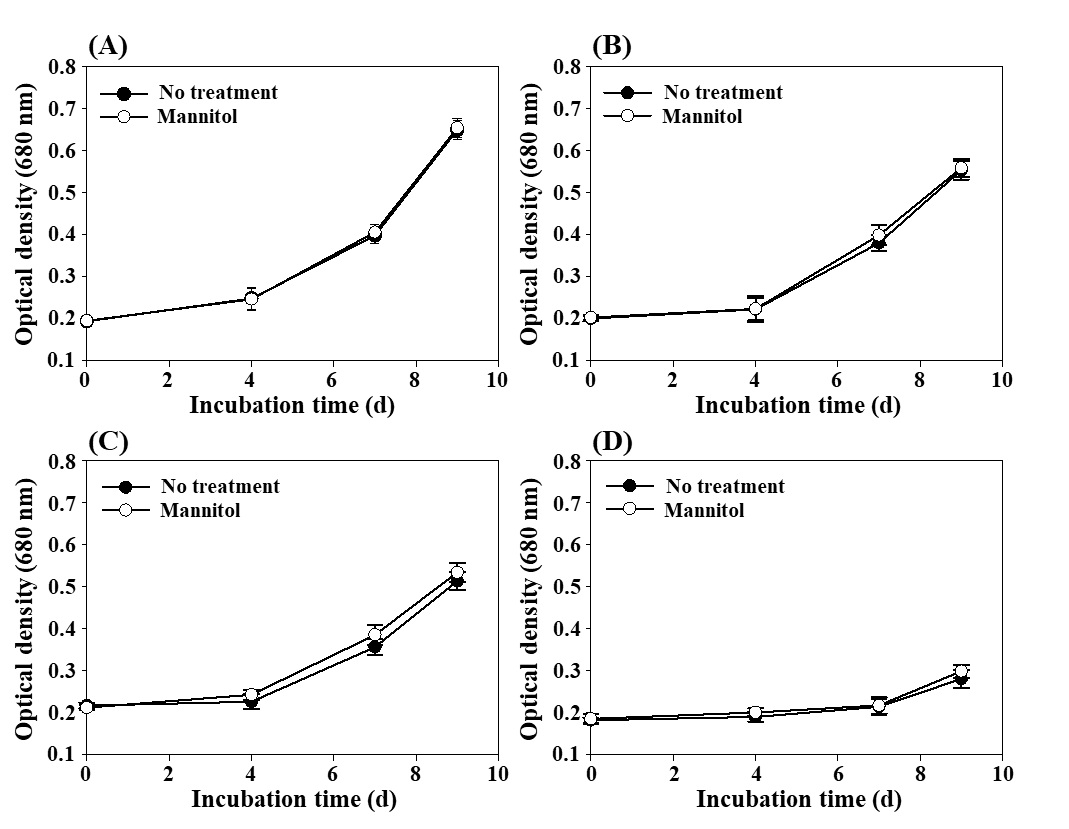


## Supplementary TABLE S1.

## Differentially expressed genes in *M. aeruginosa* NIES-298 cells treated with 25 μM and 50 μM mannitol. Only up- or down-regulated genes with more than two-fold changes (|log_2_ fold-changes| >1) in 25 μM or 50 μM mannitol-treated cells were listed. Expression levels of the genes were indicated as reads per kilobase of gene per million mapped reads (RPKM) and as fold changes (log_2_ fold-changes) relative to expression levels in control cells (untreated).

| Locus tag | Gene product | RPKM value | | |  | Fold-change **(**log_2_**)** | |
| --- | --- | --- | --- | --- | --- | --- | --- |
|  |  | Control | 25 μM  mannitol | 50 μM  mannitol |  | 25 μM  mannitol | 50 μM  mannitol |
| D3800_03175 | hypothetical protein | 7.36 | 14.12 | 17.96 |  | 0.94 | 1.29 |
| D3800_00960 | methyltransferase domain-containing protein | 40.02 | 50.43 | 93.26 |  | 0.33 | 1.22 |
| D3800_04870 | restriction endonuclease | 5.96 | 17.26 | 13.23 |  | 1.53 | 1.15 |
| D3800_14375 | hypothetical protein | 5.32 | 5.90 | 11.52 |  | 0.15 | 1.11 |
| D3800_01090 | PIN domain-containing protein | 6.33 | 5.69 | 13.14 |  | -0.15 | 1.05 |
| D3800_06740 | hypothetical protein | 12.67 | 30.58 | 26.29 |  | 1.27 | 1.05 |
| D3800_22990 | Uma2 family endonuclease | 6.39 | 7.23 | 13.09 |  | 0.18 | 1.03 |
| D3800_20305 | hypothetical protein | 5.58 | 10.32 | 11.20 |  | 0.89 | 1.01 |
| D3800_19725 | 3-oxoacyl-ACP synthase | 7.06 | 14.23 | 13.96 |  | 1.01 | 0.98 |
| D3800_16625 | hypothetical protein | 8.37 | 17.80 | 14.22 |  | 1.09 | 0.76 |
| D3800_19250 | hypothetical protein | 15.71 | 7.44 | 16.05 |  | -1.08 | 0.03 |
| D3800_03845 | hypothetical protein | 12.41 | 5.35 | 8.66 |  | -1.21 | -0.52 |
| D3800_07110 | hypothetical protein | 14.22 | 6.34 | 7.40 |  | -1.17 | -0.94 |
| D3800_23305 | DUF4926 domain-containing protein | 23.31 | 20.62 | 11.28 |  | -0.18 | -1.05 |
| D3800_05590 | transcriptional regulator | 14.95 | 10.19 | 7.21 |  | -0.55 | -1.05 |

**Supplementary TABLE S2**.

Expression of putative caspase genes identified from *M. aeruginosa* NIES-298. Expression levels of the genes were indicated as reads per kilobase of gene per million mapped reads (RPKM) and as fold changes (log_2_ fold-changes) relative to expression levels in control cells (untreated).

| Locus tag | RPKM value | | |  | Fold-change **(**log_2_**)** | |
| --- | --- | --- | --- | --- | --- | --- |
|  | Control | 25 μM mannitol | 50 μM mannitol |  | 25 μM mannitol | 50 μM mannitol |
| D3800_09480 | 28.90 | 27.39 | 25.91 |  | -0.13 | -0.27 |
| D3800_10800 | 80.70 | 83.59 | 88.48 |  | 0.09 | 0.23 |
| D3800_10885 | 31.27 | 32.34 | 40.06 |  | 0.08 | 0.61 |
| D3800_18795 | 6.20 | 4.71 | 5.94 |  | -0.68 | -0.11 |
